# Motivation and guidance of college students’ low-carbon behavior: evidence from Chinese colleges and universities

**DOI:** 10.3389/fpsyg.2024.1375583

**Published:** 2024-06-13

**Authors:** Gaofei Ren, Changjin Liu, Yaoyao Chen

**Affiliations:** ^1^School of Management, Jiujiang University, Jiujiang, China; ^2^Jiangxi Yangtze River Economic Zone Research Institute, Jiujiang, China; ^3^School of Economics and Management, Nanchang Hangkong University, Nanchang, China

**Keywords:** carbon peak and carbon neutrality, college students, low-carbon behavior, motivation and guidance, theory of planned behavior

## Abstract

In the context of the global implementation of the emission peak and carbon-neutral strategic goal, guiding residents’ low-carbon behavior is of great significance for the realization of the dual carbon goal. However, existing studies have paid less attention to the low-carbon behavior of college students. Based on the theory of planned behavior, this paper constructs a theoretical model of influencing factors of college students’ low-carbon behavior. Combined with 612 questionnaires from Chinese colleges and universities, this study uses a structural equation model and multi-group analysis method to explore the motivation of college students’ low-carbon behavior and guiding education strategies. The results show that low-carbon attitude, subjective norms, low-carbon values, and perceived behavior control have significant positive effects on low low-carbon behavior intention of college students, and influence their low-carbon behavior through low-carbon behavior intention. Further research found that gender and growth environment (urban vs. rural) presented heterogeneity in different influence paths, and the perceived cost had a significant negative moderating effect during the transition from low-carbon intention to low-carbon behavior. These research findings provide a theoretical basis and policy inspiration for explaining and guiding the low-carbon behavior of college students.

## 1 Introduction

In response to global climate change, countries worldwide have adopted the carbon peak and carbon neutrality (dual-carbon) targets as critical strategies for transitioning to a low-carbon economy. China, in its 14th Five-Year Plan, has emphasized the expansion of consumption of green and low-carbon products, the promotion of green and low-carbon lifestyles, and the enhancement of environmental awareness and ecological consciousness in the nationwide green and low-carbon action. How to guide the low-carbon behavior of the whole people to help achieve the goal of double carbon has become an important theoretical and practical problem that needs to be solved urgently. Against the backdrop of nationwide low-carbon actions, college students have emerged as one of the major end consumers in terms of energy consumption and industrial energy-intensive products. This demographic possesses significant carbon emission reduction potential through their low-carbon actions (Liu et al., 2019). Guiding the vast population of college students to transition to low-carbon lifestyles can not only promote the establishment of low-carbon cities and campuses but also accelerate the achievement of dual-carbon targets.

To cope with climate change, more than 120 countries and regions around the world have proposed the goal of dual carbon. Different scholars have carried out research on dual carbon from different perspectives. Most of existing studies have discussed the issue of dual carbon from the industry and enterprise side, these studies f found that enterprises can promote residents’ low-carbon behaviors by using green technologies and resources (Zhong et al., 2018; Grainger and Smith, 2021; Hepburn et al., 2021; Gao and Zhang, 2022). The residential field has become a hot spot in recent years (Tan et al., 2021; Zhang et al., 2021). The research on residents’ low-carbon behavior mainly focuses on the influencing factors and management countermeasures. As for the research on the influencing factors of residents’ low-carbon behavior, it has been found that environmental sensitivity, low-carbon cognition level, and low-carbon publicity have important impacts on low-carbon behavior (Gao et al., 2017; Zhang et al., 2020). In terms of the research on the management countermeasures of residents’ low-carbon behaviors, scholars have discussed the countermeasures of residents’ low-carbon behaviors from different perspectives, such as cultivating the awareness of environmental responsibility (Jiang and Jiang, 2020; Hepburn et al., 2021), guiding low-carbon travel, etc. (Moody and Zhao, 2020; Yuan et al., 2021). However, based on the two-carbon goal, the influencing factors and mechanisms of low-carbon behaviors of college students need to be further clarified.

While numerous studies have focused on low-carbon behaviors in the context of dual-carbon targets, there are still certain limitations. From a research perspective, most studies have primarily examined low-carbon production behaviors from the viewpoint of enterprises or industries, neglecting college students as a distinct demographic in studies of residential low-carbon behaviors. In terms of strategic research, while scholars have reached a consensus regarding the importance of guiding residential low-carbon behaviors (Cai et al., 2019; Hu et al., 2021), further research is needed to explore how to cultivate college students’ willingness for low-carbon behaviors and guide their actual low-carbon behaviors within the university context. College students represent a crucial reservoir for economic and social green and low-carbon development, possessing significant potential to mitigate climate change and protect the environment. Their psychological and behavioral aspects directly influence the effectiveness of constructing a green and low-carbon economy. Moreover, college students are at a pivotal stage of learning and development, during which their values and behavioral habits are often formed and can profoundly impact their future behaviors. Therefore, this study focuses on college students and delves into their low-carbon behaviors and motivations.

The novelties and theoretical contributions of this paper are as follows. Firstly, we integrate the Theory of Planned Behavior (TPB) by incorporating low-carbon values as a significant antecedent variable of behavioral intentions, alongside attitude, subjective norm, and perceived behavioral control. We explore the effects of these four factors on college students’ intentions for low-carbon behaviors, thereby addressing the oversight of the TPB model regarding the value aspect of behavioral intentions and expanding the research context of low-carbon behaviors from the perspective of college students as a demographic group. Secondly, while previous studies on low-carbon behaviors have mainly focused on the direct effects of various antecedents on low-carbon intentions and the mediating role of low-carbon intentions on low-carbon behaviors, this study incorporates perceived cost as a moderating variable and constructs a moderated mediating model, enriching the research on the mechanism of influencing factors of low-carbon behaviors. Lastly, we incorporate gender and developmental environment as individual characteristic factors in comparative studies and reveal the differential effects of gender and developmental environment on low-carbon behaviors, providing insights for future research on low-carbon behaviors.

The structure is designed as follows. Section 2 describes the theoretical basis and puts forward the research hypothesis. In section 3, variable measurements, data collection, and demographic interpretation are explained. Section 4 verifies different hypotheses through reliability and validity testing, correlation analysis and structural equation model analysis. Conclusions and policy recommendations are forward in Section 5.

## 2 Theoretical basis and research hypothesis

The TPB is widely used as a basic theory in the field of behavior research. Similar to the hypothesis of Rational Behavior Theory, individual behavior is caused by behavioral intention, which is jointly determined by the individual’s attitude toward behavior, subjective norms of behavior, and other factors. In this theory, behavioral intention is the most important determinant of behavior (Mancha and Yoder, 2015). According to the TPB, behavior is triggered by behavioral intention and behavioral control, which are usually determined by attitude, subjective norm, and perceived behavioral control and their interactions. Among them, attitude is a kind of psychological emotion, which is the evaluation of the consequences of rational choice of specific behavior. If individuals have a positive attitude toward environmental problems, they will show friendly environmental behavior. Subjective norms mean that an individual’s behavior is influenced by social pressures from others that may cause them to behave in a certain way that others follow. Perceived behavioral control is an individual’s belief in their ability to overcome obstacles and perform a given behavior (Ajzen, 1991). The TPB has been widely used to explain various behaviors, the theory has been introduced into the framework of residents’ intention to low-carbon transportation and behavior analysis (Yuan et al., 2021), and it has also been used to explain urban residents’ intention to low-carbon behavior in energy consumption. Based on the validity and simplicity of the TPB, we choose this model as the basic theoretical framework. The specific behavior types supported by personal values and normative beliefs are also affected by other factors, such as time, money, ability, and other resource constraints (Stern et al., 1999). To better interpret the formation mechanism of college students’ low-carbon behavior intention, this study proposes an improved theoretical model of TPB, and further regards low-carbon values as the antecedent variable of low-carbon behavior intention, the perceived cost is taken as the moderating variable of the transformation from low-carbon behavior intention to low-carbon behavior, to deeply explore the psychological motivation and influence mechanism of low-carbon behavior of college students.

Individual factors affecting low-carbon behavior include psychological factors (low-carbon intention, attitude, emotion, self-efficacy, etc.) and non-psychological factors (low-carbon knowledge, low-carbon behavior ability, habits, etc.), which have an impact on low-carbon consumption behavior (Liu et al., 2019). Attitude is an individual’s perception of a certain kind of behavior, which has been proven to be an important factor affecting individual behavior. The TPB proposed by Ajzen (1991) is mainly used to analyze how individual attitudes consciously affect behaviors, focusing on the formation process of behaviors based on cognitive information. When respondents rated their environmental attitudes as positive, their behavior in terms of energy use was also positive. A positive attitude helps to explain environmental protection behavior (Echegaray and Hansstein, 2017), and the more positive the low-carbon attitude, the stronger the intention for low-carbon behavior may be. Meanwhile, based on the original variables of TPB, both the social pressure (Subjective Norm) that individuals feel about whether to take a specific behavior and the perceived ability to control their behavior under certain conditions (Perceived Behavioral Control) have a positive impact on behavioral intention (Ajzen, 1991). Therefore, we propose the following research hypothesis:

*H1*: Low-carbon attitude has a significant positive effect on college students' low-carbon behavior intention.

*H2*: Low-carbon subjective norms have a significant positive effect on college students' low-carbon behavior intention.

*H3*: Low-carbon perceived behavior control has a significant positive effect on college students' low-carbon behavior intention.

Value belief is an important variable of normative activation theory, which is often used in the research field of public environmental protection behavior. Stern et al. (1999) first established the normative theory of value beliefs and conceptualized the relationship between values, beliefs, norms, and behaviors in the causal chain, these values and beliefs contribute to the development of personal environmental norms and lead to a series of behavioral changes (Sarkis, 2017). Values are the guiding principles in life and the internal determining factor for individuals to decide whether to take action, while consumer values play a crucial role in guiding individual consumption attitudes and behaviors (Chen et al., 2014). Stern et al. (1993) proposed three types of values related to environmental protection: egoistic values that promote private interests, altruistic values that consider the welfare of others, and ecological values that protect the environment. This study defines low-carbon values as encompassing a low-carbon lifestyle with sustainable development as its focus, actively adopting green and low-carbon lifestyles to reduce the adverse effects of environmental issues on oneself, others, and the environment, with the value goal of harmonious coexistence between humans and nature, based on three types of environmental values: self-interest, altruism, and environmental benefit. Some scholars have further developed the normative theory of value beliefs, linking multiple factors such as personal values, ability to reduce environmental threats, and personal norms of pro-environmental behaviors with causal chains to better explain environmental behavior indicators, because beliefs have a greater impact on low-carbon behaviors than economic variables, and the intention to take environmental action is driven by environmental concerns (Girod et al., 2017; Sarkis, 2017). When individuals hold firm beliefs and values that protecting the environment is essential, pro-environmental sentiments guide their norms, which in turn motivate them to take action to protect the environment (Davari and Strutton, 2014). Based on this, research hypothesis 4 is proposed:

*H4*: Low-carbon values have a significant positive effect on college students' low-carbon behavior intention.

Behavioral intention is the purpose and degree to which an individual strives to act for taking it. According to the TPB, individual decision or intention is the most direct and important predictor of behavior (Ajzen, 1991), Hines et al. (1987) also found that behavior intention is the direct antecedent variable of environmental behavior, and this conclusion has also been supported by the research on low-carbon behavior in recent years (Mi et al., 2019; Li W. et al., 2021; Yuan et al., 2021). Therefore, we propose research hypothesis 5:

*H5*: The intention of low-carbon behavior has a significant positive effect on college students' low-carbon behavior.

Perceived cost serves as a pivotal factor influencing individuals’ intention and actual behavior enactment (Almansoori et al., 2023; Tannady and Dewi, 2024). From a cost–benefit analysis perspective, individuals weigh the costs and benefits when deciding whether to engage in a particular behavior. If the costs associated with a behavior are perceived as too high, individuals may opt to avoid undertaking such behavior (Wu et al., 2023). In the context of low-carbon behavior, if college students perceive that adopting low-carbon practices entails elevated costs, such as additional expenditure on environmentally friendly products or altering their lifestyles, they may hesitate or choose not to adopt these behaviors. Consequently, attitudes and intentions merely represent a general inclination toward behavior. When external factors like costs are weak, attitudes and intentions are closely correlated with behavior. However, when external influences such as costs exert a stronger impact, attitudes and intentions have less influence on individual behavior (Ding et al., 2018). Due to the constraints of costs, behavioral intentions do not always translate into actual behavior. The Motivation-Ability-Opportunity model posits that, in circumstances where opportunities are not restricted, behavior is driven by both behavioral motivation and capability (Ölander and Thøgersen, 1995). Hence, the implementation of low-carbon behavior among university students depends not only on their intention but also on their ability to enact such behavior.

The theory of behavioral costs suggests that costs negatively moderate the relationship between attitudes and behavior. When the costs of behavior exceed a threshold, the influence of attitudes on behavior diminishes until it disappears (Diekmann and Preisendörfer, 2003). These costs encompass not only monetary expenses but also non-monetary costs such as time, effort, convenience loss, and comfort loss. If individuals perceive that adopting low-carbon behavior will incur additional costs, they may be inclined to avoid or reduce such behavior (Ji et al., 2023). Thus, perceived cost may attenuate the positive impact of low-carbon attitudes, subjective norms, and low-carbon values on college students’ intention to engage in low-carbon behavior, thereby negatively moderating the relationship between low-carbon intentions and behavior. Consequently, lower perception of costs regarding implementing low-carbon behavior increases the likelihood of translating low-carbon behavioral intentions into actual actions. Therefore, hypothesis 6 is proposed:

*H6*: Perceived cost has a significant negative moderating effect on college students’ low-carbon behavior intention to transform into low-carbon behavior.

**Figure 1:**
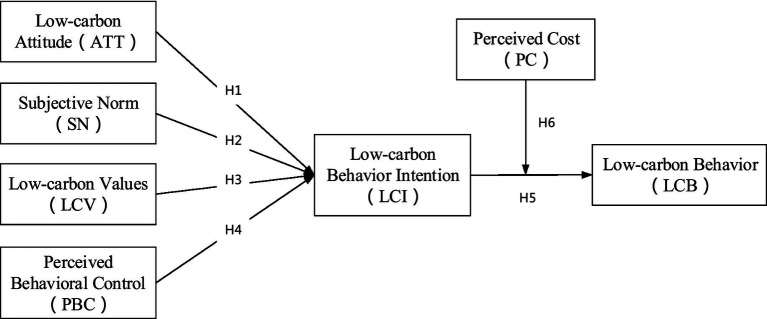
Theoretical model of low carbon behavior motivation of college students.

In summary, this paper proposes a theoretical model of psychological motivation for college students’ low-carbon behavior, as shown in Figure 1.

## 3 Research design

### 3.1 Variable measurement

The variables in this study were measured using a 5-level Likert scale, with a value of 1–5 according to the increasing degree (1 means completely disagree, 5 means completely agree). In the process of compiling the scale, the measurement of low-carbon attitude (ATT) refers to the scale of Haustein and Hunecke (2007); Subjective norm (SN) and perceived behavioral control (PBC) were measured according to Klöckner and Matthies (2009); Low carbon values (LCV) and low carbon behaviors (LCB) refer to the research of Mi et al. (2016); The measurement of low carbon behavioral intention (LCI) was based on the studies of Ajzen (1991), Stern et al. (1999) and Chan (2001), and revised according to the subjects; Perceived cost (PC) draws on the research of Kim et al. (2007) and Yang et al. (2010), and which is modified according to the content of this study. Before the formal investigation, the initial scale was pre-investigated. Through exploratory factor analysis, reliability, and validity tests, items that did not meet the test criteria in the initial scale were modified and deleted. The final formal scale contained 24 items (Table 1).

**Table 1:** Descriptive statistics of main variables.

Variable	N	Min	Max	Mean	SD
ATT	612	3	15	7.949	3.071
SN	612	3	15	10.811	2.607
LCV	612	4	15	9.224	2.534
PBC	612	4	20	12.778	3.923
LCI	612	4	20	11.953	3.593
PC	612	3	15	8.899	2.532
LCB	612	4	20	12.717	3.294

### 3.2 Data collection

The formal survey of this study took college students as the respondent, and the questionnaire was administered by research team members after obtaining approval from the ethics committee. The survey was conducted twice, in September and November 2022, at 9 universities in Nanchang and Jiujiang. During the survey, first, the investigators explained the specific meanings of the variables to the respondents in writing; second, after obtaining informed consent, the respondents were asked to truthfully complete the questionnaire based on their feelings or thoughts. To ensure the authenticity and reliability of the research data, anonymous surveys were conducted, and the true purpose of the survey was disclosed to the respondents after the completion of the questionnaire. After sample verification and data correction, a total of 612 valid questionnaires were finally obtained.

In the questionnaire design and data collection process, this study controlled for common method bias by expanding the sources of questionnaire collection, improving the scale items, and conducting anonymous surveys. However, it may still not eliminate the problem of common method bias due to consistent sample background and fixed format of the scale items. Therefore, this study used the Harman single-factor test to examine the potential common method bias issue. The results showed that the unrotated first principal component factor explained 40.564% of the variance, which is less than 50% (Podsakoff et al., 2003), indicating that the common method bias in the data is within an acceptable range. Descriptive statistics of the sample and main variables are presented in Tables 1, 2, respectively.

**Table 2:** Demographics characteristics of participants.

Demographics	Categories	Frequency	Percent (%)
Gender	Male	318	52%
	Female	294	48%
Age	<17	61	10%
	18–21	449	73%
	>21	102	17%
Grade	Fresh man	226	37%
	Sophomore	185	30%
	Junior	133	22%
	Senior	68	11%
Major field	Arts	233	38%
	Science	183	30%
	Engineering	196	32%
Growth environment	Urban	215	35%
	Rural	397	65%

## 4 Empirical analysis

### 4.1 Reliability and validity test

Firstly, Cronbach’s α was used to test the reliability of the scale, and its convergence validity was measured by the Average Variance Extracted (AVE) of each latent variable, and then the discriminant validity was tested by comparing the correlation coefficient of each factor and AVE square root. The analysis results showed that Cronbach’s α values of seven potential variables, including low-carbon attitude, subjective norms, perceived behavior control, low-carbon values, low-carbon behavior intention, perceived cost, and low-carbon behavior, were all greater than the standard value 0.7 (Table 3), indicating that the scale had good reliability.

**Table 3:** Confirmatory factor analysis and reliability test results.

Variable	Coding	Item	Factor loading	Cronbach’s α	AVE
Low-carbon Attitude (ATT)	ATT1	It is necessary and significant to advocate the low-carbon behavior of college students	0.917	0.896	0.827
	ATT2	Low-carbon behaviors such as buying low-carbon products can alleviate environmental problems	0.918		
	ATT3	It is the responsibility and obligation of every college student to practice low-carbon behavior	0.893		
Subjective Norms (SN)	SN1	My low-carbon behavior is influenced by my family, friends, and classmates	0.869	0.816	0.733
	SN2	High energy consumption will be criticized by family, friends, and classmates	0.882		
	SN3	I feel honored to participate in low-carbon energy-saving activities	0.816		
Low-carbon Values (LCV)	LCV1	Low-carbon behavior does not limit my personal choice and freedom	0.800	0.773	0.689
	LCV2	Individual low-carbon behavior will have a positive impact on others	0.877		
	LCV3	Low-carbon behavior is conducive to protecting the ecological environment	0.811		
Perceived Behavior Control (PBC)	PBC1	Low-carbon labels such as green products will drive me to buy low-carbon and energy-saving products	0.753	0.841	0.677
	PBC2	I can easily buy low-carbon and energy-saving products	0.834		
	PBC3	I can skillfully use bicycles, shared bikes, and other low-carbon transportation	0.862		
	PBC4	It’s entirely up to me whether or not I implement low-carbon behaviors	0.839		
Low-carbon Behavior Intention (LCI)	LCI1	I am willing to buy low-carbon and energy-saving products	0.868	0.888	0.749
	LCI2	I would like to use low-carbon transportation	0.868		
	LCI3	I’m willing to turn off appliances when not in use to reduce standby power consumption	0.874		
	LCI4	I am willing to be a volunteer for low-carbon energy-saving propaganda on campus	0.851		
Perceived Cost (PC)	PC1	I think the current implementation of low-carbon behavior needs to bear a high cost	0.860	0.815	0.731
	PC2	I think the high cost is the barrier to purchase and use low-carbon products	0.862		
	PC3	I think low-carbon products may not meet the promised energy-saving and environmental protection effects	0.843		
Low-carbon Behavior (LCB)	LCB1	I often use public transportation, such as busses, bikes, and so on	0.817	0.791	0.632
	LCB2	I will take the initiative to buy all kinds of green and low-carbon products	0.877		
	LCB3	If I’m the last one to leave my dorm or classroom, I will turn off the lights	0.888		
	LCB4	I also activate sleep mode when I do not use the computer for short periods	0.550		

The validity test results of the scale show that the standard factor load coefficients of all latent variables are between 0.6 and 0.9, and the AVE value is greater than the standard value of 0.5 (Table 1), and the convergence validity is good. The AVE square root of each latent variable is greater than the correlation coefficient of each latent variable (Table 4), indicating that the variables in the scale have good discriminant validity.

**Table 4:** Discriminant validity test results.

Variable	ATT	SN	LCV	PBC	LCI	PC	LCB
ATT	0.909						
SN	0.540	0.856					
LCV	0.574	0.463	0.830				
PBC	0.689	0.495	0.443	0.823			
LCI	0.740	0.609	0.573	0.654	0.865		
PC	0.363	0.373	0.289	0.295	0.515	0.855	
LCB	0.560	0.606	0.434	0.544	0.665	0.448	0.795

The diagonal value is the square root of AVE, and the remaining value is the correlation coefficient between the variables.

### 4.2 Path analysis

After the reliability and validity tests of the measurement models, AMOS 24 software’s Maximum Likelihood Estimation is used to fit and analyze the structural equation models. The fitting results (Table 5) show that each test index meets the fitting criteria (Joreskog and Sorbom, 1993), indicating that the model is a good fit for the sample data. The path coefficient (Table 6) shows that college students’ low carbon attitude (β = 0.372, *p* < 0.001), subjective norm (β = 0.285, *p* < 0.001), low carbon values (β = 0.174, *p* < 0.001), and perceived behavior control (β = 0.201, *p* < 0.001). *p* < 0.001 had a significant positive driving effect on low-carbon behavior intention and acted on low-carbon behavior through low-carbon behavior intention (β = 0.763, *p* < 0.001). Therefore, hypotheses H1, H2, H3, H4, and H5 are all supported. The influencing factors of low-carbon intention are low-carbon attitude, subjective norm, perceived behavior control, and low carbon values in order of path coefficient, indicating that low-carbon attitude and subjective norm are the main factors for college students to generate low-carbon behavior intention. In the structural model test, the gender and growth environment of the surveyed college students were taken as the control variables affecting their low-carbon behavior. The standardized path coefficient showed that gender (β = 0.065, *p* < 0.05) and growth environment (β = 0.059, *p* < 0.1) both had significant effects on low-carbon behavior. Therefore, a multi-group analysis will be further conducted based on these two individual characteristics.

**Table 5:** The results of the overall fitting of the model.

Test index	CMIN/DF	GFI	AGFI	NFI	CFI	IFI	RMSEA
Criterion	<5.000	>0.900	>0.900	>0.900	>0.900	>0.900	<0.050
Research model	4.451	0.914	0.917	0.918	0.912	0.912	0.045

**Table 6:** Structural equation model fitting results.

Variable path	Path coefficient	S.E.	C.R.	Test results
ATT → LCI	0.372^***^	0.057	5.742	H1 supported
SN → LCI	0.285^***^	0.044	6.718	H2 supported
LCV → LCI	0.174^***^	0.053	3.836	H3 supported
PBC → LCI	0.201^***^	0.066	3.627	H4 supported
LCI → LCB	0.763^***^	0.043	15.665	H5 supported

***p < 0.01, **p < 0.05, *p < 0.1.

### 4.3 Multi-group analysis of individual characteristic factors

Gender and growth environment differences may moderate the formation and transformation of intention and behaviors toward low-carbon actions by influencing individuals’ social identity, environmental self-identity, social interactions, and consumption behaviors (Li Y. et al., 2021). To further test whether there are differences among individual characteristic factors in each path of the model, based on previous studies (Gundala et al., 2022), this study conducted a multi-group analysis of gender and growth environment respectively, and the results are shown in Table 7. Compared with the main model, the influence of subjective norms on low-carbon behavior intention of male college students is not significant, and other paths are significant. First of all, gender difference has a certain moderating effect on the formation and transformation of low-carbon behavior intention. In terms of the influence of low-carbon behavior intention, the promoting effect of low-carbon attitude, low-carbon values, and perceived behavior control of male college students is more obvious, and the promoting effect of subjective norms of female college students is more obvious. In the transformation from low-carbon intention to low-carbon behavior, male college students are significantly higher than female students. Secondly, the difference in growth environment also has a certain moderating effect on the formation and transformation of low-carbon behavior intention. In terms of the impact of low-carbon behavior intention, the low-carbon attitude, low-carbon values and perceived behavior control of college students growing up in rural areas have more obvious promoting effects, while the subjective norms of college students growing up in urban areas have a more significant promoting effect. In terms of the transformation from low-carbon intention to low-carbon behavior, college students growing up in rural areas are significantly higher than those growing up in urban areas.

**Table 7:** Multi-group analysis results.

Variable path	Gender		Growth environment	
	Male student	Female student	Rural areas	Urban areas
ATT → LCI	0.453^***^	0.280^***^	0.430^***^	0.305^**^
SN → LCI	0.068	0.441^***^	0.232^***^	0.332^***^
LCV → LCI	0.232^***^	0.149^*^	0.208^**^	0.176^**^
PBC → LCI	0.269^**^	0.174^**^	0.200^**^	0.186^**^
LCI → LCB	0.788^***^	0.752^***^	0.819^***^	0.754^***^

***p < 0.01, **p < 0.05, *p < 0.1.

### 4.4 Moderating effect test

Before testing the moderating effect of perceived cost (PC), the two variables of low carbon behavioral intention (LCI) and perceived cost were normalized. Then, three models were constructed by hierarchical regression. Model 1 only included the independent variable low-carbon behavior intention and dependent variable low-carbon behavior; Model 2 adds the perceived cost of the moderating variable; based on Model 2, Model 3 adds the interaction term (LCI × PC), and judges the moderating effect based on the change of R^2^ of the determination coefficient of Model 3 compared with R^2^ of models 1 and 2 and the significance of the interaction term, the hierarchical regression results are shown in Table 8. As can be seen from Table 7, for the low carbon behavior of college students, after the addition of moderating variables and interaction terms, the determination coefficient R^2^ of model 3 is significantly higher than that of models 1 and 2, and the coefficient of interaction terms is significant (β = −0.061, *p* < 0.05), which indicates that perceived price has a significant negative moderating effect on the transformation of low-carbon behavior intention to low-carbon behavior. Therefore, hypothesis H6 is supported.

**Table 8:** Hierarchical regression results of low carbon behavior intention and perceived cost.

Independent variable	Dependent variable (LCB)		
	Model 1	Model 2	Model 3
LCI	0.665^***^	0.591^***^	0.586^***^
PC		0.144^***^	0.150^***^
LCI × PC			−0.061^**^
*R* ^2^	0.442	0.457	0.464
*F*	483.340^***^	256.712^***^	175.460^***^

***p < 0.01, **p < 0.05, *p < 0.1.

## 5 Conclusions and implications

### 5.1 Conclusion

Based on the theory of planned behavior and the three influencing factors of the original theoretical model, this study proposes a theoretical framework for the psychological motivation of college students’ low-carbon behavior by including two new variables, low-carbon values and perceived cost. Two conclusions were obtained through fitting and multi-group analysis of the sample data using a structural equation model.

Firstly, the low-carbon behavior of college students comprises two stages: the formation of intention and the transformation of intention. In the stage of forming the intention for low-carbon behavior, low-carbon attitude stands as the foremost driving factor, followed by subjective norms. Additionally, perceived behavioral control and low-carbon values also play driving roles in low-carbon intention, aligning with existing research on the influence of environmental attitudes, social pressures, and values on individuals’ environmental behavioral intentions (Chwialkowska et al., 2020; Ahmed et al., 2021; Yang et al., 2021). This suggests that college students’ intention for low-carbon behavior primarily stems from subjective attitudes toward low-carbon practices, as well as adherence to group behavior and societal norms, and value judgments. In the transition from intention to actual low-carbon behavior, perceived costs exhibit a significant negative moderating effect, consistent with prevailing viewpoints in intention-behavior research (El-Said, 2020), indicating that perceived cost factors during the implementation of environmental behaviors can influence the effectiveness of intention-behavior conversion, forming a synergistic effect. Given the relatively limited behavior and economic capacity of college students, if the implementation difficulty of low-carbon behavior is low and the prices of low-carbon products are comparatively favorable, the reduction of perceived costs can promote the conversion from low-carbon intention to behavior, effectively expanding the TPB and existing research findings, while providing new insights for governments and universities in formulating low-carbon guidance policies.

Secondly, individual characteristics exhibit heterogeneity in the formation and transformation of intention for low-carbon behavior among college students. Regarding gender, male college students demonstrate a greater driving effect of low-carbon attitude, low-carbon values, and perceived behavioral control on low-carbon intention, whereas subjective norms exert a greater driving effect on low-carbon intention among female college students. In the transition from intention to behavior, male college students significantly outperform females, these conclusions reaffirm the moderating role of gender factors in environmental behavior (Ahmad et al., 2021). At the level of growth environment, college students raised in rural areas show a greater driving effect of low-carbon attitude, low-carbon values, and perceived behavioral control on low-carbon intention, whereas subjective norms have a greater driving effect on low-carbon intention among those raised in urban areas. In the transition from intention to behavior, college students raised in rural areas significantly surpass those raised in urban areas. These conclusions further validate the heterogeneity of individual characteristics in environmental behavior (Soutter et al., 2020; Carrus et al., 2021). These findings comprehensively reflecting the influence modes and paths of individual gender and growth environment on the formation and transformation of intention for low-carbon behavior. This not only broadens the perspective of low-carbon behavior research but also holds certain enlightening significance for studies on environmental behavioral group differences.

### 5.2 Implications

#### 5.2.1 Cultivate low-carbon attitudes and values of college students

As the main place for college students to live and study, colleges and universities have an inescapable responsibility and obligation for the low-carbon education and value cultivation of college students. To cultivate college students’ positive low-carbon attitudes and values and guide their low-carbon consumption, colleges and universities should combine double carbon-related industrial policies to carry out low-carbon policy publicity and education, set up general education courses on environmental protection, and encourage teachers to integrate low-carbon education into course teaching. Especially on special festivals such as World Environment Day, low-carbon knowledge popularization education is carried out through various themed activities to shape the low-carbon values of college students. Schools should strengthen the green and low-carbon infrastructure, formulate regulations on energy conservation and emission reduction, and establish corresponding feedback mechanisms to improve the participation rate of various low-carbon activities. Cultivate students’ low-carbon living, learning, and consumption habits, and enhance their sense of honor and responsibility in practicing low-carbon behaviors by establishing low-carbon models in dormitories, canteens, and classrooms. Meanwhile, colleges and universities should put up low-carbon signs and specifications in campus public places to encourage students to realize the harm of high carbon, to form low-carbon social norms, and to create a herd mentality of low-carbon behavior on campus. Relying on community practice activities to guide students’ low-carbon consumption, actively participate in ecological public welfare activities, improve students’ perceptual behavior control with strong institutional norms, and transform low-carbon intention into low-carbon behavior.

#### 5.2.2 Policy design based on gender and growth environment differences among college students

At present, few studies have focused on the heterogeneous effects of gender and growth environment factors on the formation and transformation of low-carbon behavior intention of college students. By incorporating the heterogeneity of college students’ characteristics into the formulation of low-carbon behavior guidance policies, different policies and practices can help enhance the formation of college students’ low-carbon intention and the transformation of low-carbon behavior. Given the weak driving effect of low-carbon attitude, low-carbon values, and perceived behavior control on low-carbon intention of female students and college students from urban areas, and the low conversion rate of low-carbon intention, schools can focus on strengthening low-carbon education for majors with a high proportion of female students and college students from urban areas in the process of publicity and education on low-carbon knowledge such as water and electricity saving. The incomplete cognition of these college students on low-carbon knowledge and policies should be changed through videos, live demonstrations, and practical activities, to promote the formation of low-carbon attitudes and values, improve their ability to control low-carbon perceptual behaviors, strengthen their sense of responsibility and urgency to practice low-carbon behaviors.

#### 5.2.3 Reduce the perceived cost of low-carbon behavior

The perceived cost of college students plays a negative moderating role in the influence of low-carbon behavior intention on low-carbon behavior. Therefore, reducing the perceived cost of college students’ low-carbon behavior will help them transform their low-carbon intention into low-carbon behavior. The economic cost, learning cost, time cost, and psychological cost of implementing low-carbon behavior all belong to the category of perceived cost. In terms of economic costs, the government should give certain preferential treatment to products that meet low-carbon standards in terms of taxation, policies, and market access, reduce the price of green and low-carbon products through taxation mechanisms, and correspondingly include high-carbon products or industries in the scope of taxation such as consumption tax and environmental protection tax, and raise their relative prices through tax costs. The government’s price subsidies for low-carbon products or consumers are also an effective way to enhance the purchase intention and ability of low-carbon products, especially for college students to buy energy-saving products to give price subsidies and to stimulate their purchase of low-carbon learning and living products. Meanwhile, colleges and universities should strengthen the push and display of low-carbon knowledge through various Internet platforms, apps, and publicity windows on campus, such as the use of dormitory air conditioning energy-saving mode, the time setting of computer sleep mode, and the habit education of the last student leaving the dormitory or classroom to turn off the lights, etc., to reduce the learning cost, time cost and psychological cost of low-carbon behavior of college students, and to increase the conversion rate of low-carbon intentions and the participation rate of low-carbon behavior.

## Data availability statement

The datasets presented in this study can be found in online repositories. The names of the repository/repositories and accession number(s) can be found at: https://www.jianguoyun.com/p/DcA51BYQg4_JBhj5tscFIAA.

## Ethics statement

The studies involving humans were approved by Ethics Committee of Management School, Jiujiang University. The studies were conducted in accordance with the local legislation and institutional requirements. Written informed consent for participation in this study was provided by the participants’ legal guardians/next of kin.

## Author contributions

GR: Writing – review & editing, Writing – original draft, Project administration, Methodology, Investigation, Funding acquisition, Formal analysis, Data curation, Conceptualization. CL: Writing – review & editing, Funding acquisition, Data curation. YC: Writing – review & editing, Writing – original draft, Investigation.
