# Supplementary material for: Motivation and guidance of college students’ low-carbon behavior: evidence from Chinese colleges and universities
Source: Front Psychol. 2024 Jun 13;15:1375583. doi: 10.3389/fpsyg.2024.1375583 (PMC11232552; doi:10.3389/fpsyg.2024.1375583)
Supplement: Supplementary file 1 [file Data_Sheet_1.pdf]

Motivation and guidance of college students' low-carbon behavior: Evidence from Chinese colleges and universities Questionnaire

First of all, thank you for taking the time to help us complete the paper cross-section data acquisition. This is an academic questionnaire to explore the low carbon behavior willingness of college students. This questionnaire is anonymous, and the survey objects are college students. The survey results are only used for academic research, without any commercial purpose. There is no right or wrong answer to the questionnaire, please choose the answer according to your actual experience.

Thank you again for your help and support!

● Low-carbon Attitude (ATT)

1. ATT1: It is necessary and significant to advocate the low-carbon behavior of college students

○Strongly disagree ○Disagree ○Not sure ○Agree ○Strongly agree

2. ATT2: Low-carbon behaviors such as buying low-carbon products can alleviate environmental problems

○Strongly disagree ○Disagree ○Not sure ○Agree ○Strongly agree

3. ATT3: It is the responsibility and obligation of every college student to practice low-carbon behavior

○Strongly disagree ○Disagree ○Not sure ○Agree ○Strongly agree

● Subjective Norms (SN)

4. SN1: My low-carbon behavior is influenced by my family, friends, and classmates

○Strongly disagree ○Disagree ○Not sure ○Agree ○Strongly agree

5. SN2: High energy consumption will be criticized by family, friends, and classmates

○Strongly disagree ○Disagree ○Not sure ○Agree ○Strongly agree

6. SN3: I feel honored to participate in low-carbon energy-saving activities

○Strongly disagree ○Disagree ○Not sure ○Agree ○Strongly agree

● Low-carbon Values (LCV)

7. LCV1: Low-carbon behavior does not limit my personal choice and freedom

○Strongly disagree ○Disagree ○Not sure ○Agree ○Strongly agree

8. LCV2: Individual low-carbon behavior will have a positive impact on others

○Strongly disagree ○Disagree ○Not sure ○Agree ○Strongly agree

9. LCV3: Low-carbon behavior is conducive to protecting the ecological environment
○Strongly disagree ○Disagree ○Not sure ○Agree ○Strongly agree

- **Perceived Behavior Control (PBC)**

10. PBC1: Low-carbon labels such as green products will drive me to buy low-carbon and energy-saving products
○Strongly disagree ○Disagree ○Not sure ○Agree ○Strongly agree
11. PBC2: I can easily buy low-carbon and energy-saving products
○Strongly disagree ○Disagree ○Not sure ○Agree ○Strongly agree
12. PBC3: I can skillfully use bicycles, shared bikes, and other low-carbon transportation
○Strongly disagree ○Disagree ○Not sure ○Agree ○Strongly agree
13. PBC4: It's entirely up to me whether or not I implement low-carbon behaviors
○Strongly disagree ○Disagree ○Not sure ○Agree ○Strongly agree

- **Low-carbon Behavior Intention (LCI)**

14. LCI1: I am willing to buy low-carbon and energy-saving products
○Strongly disagree ○Disagree ○Not sure ○Agree ○Strongly agree
15. LCI2: I would like to use low-carbon transportation
○Strongly disagree ○Disagree ○Not sure ○Agree ○Strongly agree
16. LCI3: I'm willing to turn off appliances when not in use to reduce standby power consumption
○Strongly disagree ○Disagree ○Not sure ○Agree ○Strongly agree
17. LCI4: I am willing to be a volunteer for low-carbon energy-saving propaganda on campus
○Strongly disagree ○Disagree ○Not sure ○Agree ○Strongly agree

- **Perceived Cost (PC)**

18 PC1: I think the current implementation of low-carbon behavior needs to bear a high cost
○Strongly disagree ○Disagree ○Not sure ○Agree ○Strongly agree
19. PC2: I think the high cost is the barrier to purchase and use low-carbon products
○Strongly disagree ○Disagree ○Not sure ○Agree ○Strongly agree
20. PC3: I think low-carbon products may not meet the promised energy-saving and environmental protection effects
○Strongly disagree ○Disagree ○Not sure ○Agree ○Strongly agree

- **Low-carbon Behavior (LCB)**

21. LCB1: I often use public transportation, such as buses, bikes, and so on
○Strongly disagree ○Disagree ○Not sure ○Agree ○Strongly agree
22. LCB2: I will take the initiative to buy all kinds of green and low-carbon products
○Strongly disagree ○Disagree ○Not sure ○Agree ○Strongly agree
23. LCB3: If I'm the last one to leave my dorm or classroom, I will turn off the lights
○Strongly disagree ○Disagree ○Not sure ○Agree ○Strongly agree
24. LCB4: I also activate sleep mode when I don't use the computer for short periods
○Strongly disagree ○Disagree ○Not sure ○Agree ○Strongly agree

- **Basic personal information**

25. GENDER: Your gender is?
○Male ○Female
26. AGE: Your age is?
○Under 17 ○18-21 ○More than 21
27. Grade: What grade are you in now?
○Fresh man ○Sophomore ○Junior ○Senior
28. Major Field: Your major field is?
○Arts ○Science ○Engineering
29. Growth Environment: Is your hometown in the city or the countryside?
○City ○Countryside
